# The role of leptin in selected skin diseases

**DOI:** 10.1186/s12944-020-01391-8

**Published:** 2020-10-02

**Authors:** Klaudia Dopytalska, Agnieszka Baranowska-Bik, Marek Roszkiewicz, Wojciech Bik, Irena Walecka

**Affiliations:** 1 Department of Dermatology, Centre of Postgraduate Medical Education, Woloska 137, 02-507 Warsaw, Poland; 2 Department of Endocrinology, Centre of Postgraduate Medical Education, Ceglowska 80, 01-809 Warsaw, Poland; 3 Department of Neuroendocrinology, Centre of Postgraduate Medical Education, Marymoncka 99/103, 01-813 Warsaw, Poland

**Keywords:** Leptin, Obesity, Psoriasis, Skin disease, Immune system

## Abstract

Leptin is an adipokine, adipocyte-derived compound, which acts both as a hormone and cytokine. It is mainly synthesized by adipocytes of white adipose tissue. Leptin possesses pleiotropic functions including, among others, stimulation of angiogenesis and production of proinflammatory cytokines. The various types of leptin activity are related to the wide distribution of leptin receptors. This adipokine acts by activating intracellular signaling cascades such as JAKs (Janus kinases), STATs (signal transducers and activators of transcription), and others.

In a course of obesity, an increased serum level of leptin coexists with tissue receptor resistance. It has been reported that enhanced leptin levels, leptin receptor impairment, and dysfunction of leptin signaling can influence skin and hair. The previous studies revealed the role of leptin in wound healing, hair cycle, and pathogenesis of skin diseases like psoriasis, lupus erythematosus, and skin cancers. However, the exact mechanism of leptin’s impact on the skin is still under investigation. Herein, we present the current knowledge concerning the role of leptin in psoriasis and selected skin diseases.

## Introduction

It’s an indisputable fact that the prevalence of obesity in modern society has risen significantly in the last two decades leading to an increased number of obesity-related pathological conditions [1]. Adipose tissue is energy storage and is also considered as the endocrine organ that can produce and secrete hormones and cytokines, named adipokines. Many studies have shown the role of adipokines in energy homeostasis, metabolism, endocrine and immunological activity, and, recently, also dermatological diseases.

Leptin is a 16-kDa protein, a product of the obese (ob) gene located on chromosome 7q31.3, and is synthesized and secreted by white adipose tissue. The peripheral leptin level is highly correlated with total fat mass and body mass index (BMI). It is believed that leptin is the main regulator of food intake, body mass, and metabolism. Due to a wide distribution of leptin receptors (LepRs), leptin possesses pleiotropic functions including stimulation of angiogenesis, modulation of the hormonal system, and augmentation of production of pro-inflammatory cytokines [2–6].

It has been widely suggested that many skin diseases are associated with metabolic disturbances including metabolic syndrome and obesity. Amongst these dermatological diseases, there are psoriasis, lichen planus, connective tissue diseases, bullous diseases, vitiligo, and chronic urticaria [6].

However, the role of leptin in the pathogenesis of skin diseases with the particular concern of psoriasis is still investigated. According to recent studies, leptin may influence skin pathophysiology and, consequently, might have an impact on skin diseases and systemic autoimmune disorders [2–6]. Taking into account the growing number of obese individuals all over the world and unquestionable participation of adiposity in many pathological processes, it could be presumed that the increase in body fat along with enhanced leptin secretion results in the disruption of normal processes in the skin in the majority of patients. Therefore, in this review, we discuss the mechanisms of leptin action within the skin and skin appendages. Herein, we also aim to present the current knowledge concerning the role of enhanced leptin levels in the pathomechanisms of psoriasis and selected skin diseases.

## Obesity and skin diseases

Obesity exerts a significant metabolic effect and is considered to be a state of chronic, low-grade inflammation leading to systemic consequences related to the disturbed secretion of hormones and cytokines including leptin, adiponectin, and chemokines that regulate inflammation [1, 7]. Adiposity leads to adipocyte dysfunction, increased systemic levels of proinflammatory cytokines and adipokines e.g. tumor necrosis factor α (TNF-α), interleukin (IL)-6, leptin, visfatin, resistin, angiotensin II, and plasminogen activator inhibitor 1, as well as activation of the proinflammatory signaling. Leptin stimulates the production of IL-1, IL-6, IL-12, and TNFα by innate immune cells and enhances reactive oxygen species (ROS) production [1, 7–10].

The systemic consequences of excessive adiposity also affect the skin and result in alterations in skin physiology. Therefore, it could be suggested that obesity is a risk factor for the development of many dermatological diseases. Indeed, obesity is often associated with venous stasis, lymphedema, increased rate of infection including candidiasis, intertrigo, candida folliculitis, furunculosis, erysipelas, cellulitis, erythrasma, tinea cruris, folliculitis, and necrotizing fasciitis. It also increases the risk of selected inflammatory dermatoses, in particular psoriasis, hidradenitis suppurativa, and atopic dermatitis [11, 12]. Moreover, some skin abnormalities such as acanthosis nigricans, keratosis pilaris, striae diseases, skin tags, and palmoplantar keratodermas are more often observed in obese patients than in those with normal body weight. The increased amount of androgens, insulin, growth hormone, and insulin-like growth factor in a course of obesity leads to the escalation of sebum production, which could exacerbate acne. Therefore, immune dysregulation and elevated levels of proinflammatory cytokines and adipokines, in particular leptin, have a significant influence on the skin and dermatological diseases [11, 12].

## Leptin - the mechanism of action

Leptin acts as a pleiotropic hormone and activator of the cytokine cascade. It is secreted mainly by white adipose tissue. However, smaller amounts are also produced by the hypothalamus, pituitary, gastric mucosa, bone marrow, mammary epithelium, skeletal muscle, and placenta [4]. Its secretion is increased in adiposity. Insulin, glucose, estrogens, and cytokines such as TNF-α and IL-6 might also enhance leptin secretion. Interestingly, the peripheral leptin level follows a circadian rhythm with a peak level seen at night. Furthermore, leptin concentration is correlated with fat tissue amount and BMI, and, consequently, obesity is characterized by increased leptin levels [1]. Moreover, high serum leptin concentrations coexist with leptin receptors resistance, and these disturbances are related to obesity [13].

The wide distribution of leptin receptors suggests the pleiotropic function of leptin. In detail, the expression of LepR has been found in the hypothalamus, fibroblasts, endothelial cells, keratinocytes, adipocytes, and blood mononuclear cells. LepR is a transmembrane receptor, similar to the class I cytokine receptors family. Due to the differences in the receptor structure, several forms of LepR could be distinguished: the short isoforms - LepRa, LepRc, LepRd, and LepRf; a soluble receptor (sOBR), LepRe, and LepRb which is a full-length isoform [4, 5, 14, 15]. The latter isoform is considered to be responsible for controlling food intake and energy balance. Short isoforms, LepRa and LepRc, are located predominantly in microvessels of the central nervous system, where they could be responsible for adequate leptin circulation in the cerebrospinal fluid as well as the receptor-mediated transport of leptin through the blood-brain barrier. LepRa and LepRb isoforms in the extra-neural tissues determine the functional pleiotropy of leptin, whereas the soluble form LepRe/sOBR provides binding variety and the bioavailability of leptin [4, 5, 14–17].

Leptin stimulates multiple signaling pathways including the kinase-signal transducer and activator of transcription (JAK/STAT), phosphoinositide 3-kinase (PI3K), mitogen-activated protein kinase (MAPK), extracellular signal-regulated kinase 1/2 (ERK1/2), adenosine monophosphate kinase (AMPK), and PPAR gamma coactivator/peroxisome proliferator-activated receptor (PGC /PPAR) [4, 5, 14, 16, 17]. The JAK/STAT signal transduction cascade is the main signaling pathway activated by leptin. After binding of leptin to the long isoform of the receptor, phosphorylation of Janus kinase (JAK2) is activated, subsequently prompting phosphorylation and activation of signal transducers as well as activator of transcription (STAT3). Activation of STAT3 results in dimerization, followed by migration to the nucleus, where STAT3 influences the expression of target genes such as the suppressor of cytokine signaling 3 (SOCS3). Furthermore, leptin impacts mitochondrial metabolism as it increases electrons flow, the efficiency of an oxidation-reduction reaction, and energy utilization [2, 4, 5, 14, 17–19].

## Leptin - the main functions

Under physiological conditions, leptin is secreted to limit food intake, control body mass, and stimulate energy expenditure by negative feedback at the hypothalamic nuclei. However, adiposity leads to an irrepressible increase in circulating leptin. Farther, obesity is not related to the suppression of appetite nor body mass reduction, due to leptin receptor resistance. This phenomenon might be a consequence of the dysfunction of leptin signaling pathways, limited access to the receptors, followed by changes in leptin receptor expression or signal transduction [2, 3, 13].

Besides, leptin also affects the immune system, mainly acting as a pro-inflammatory factor. Indeed, leptin activates the secretion of pro-inflammatory cytokines, as well as increases nitric oxide (NO) release and stimulates phagocytosis on monocytes/macrophages [20, 21]. In neutrophils, this adipokine induces the synthesis of oxygen free radicals. Moreover, leptin enhances cytotoxicity and proliferation of NK (natural killer) cells. It is also able to activate chemotaxis of eosinophils, basophils, and neutrophils. Besides, this adipokine increases IL-8, IL-12, IL-6, and TNF-α release from the dendritic cells. Furthermore, by influencing lymphocyte receptors, leptin affects the Th1/Th2 balance toward Th1 response and leads to an aggravation of inflammatory processes. The wide impact of leptin on the immune system could imply the important role of this adipokine in the pathogenesis of autoimmune diseases [20–22].

The animal models, especially those with impaired leptin signaling, are widely used in diabetes and obesity research. The autosomal recessive ob/ob mouse has a mutation in the leptin gene on chromosome 6 resulting in the deficiency of leptin. The phenotype of ob/ob mice includes marked obesity due to hyperphagia. Furthermore, there is also increased efficiency of energy utilization with the rate of lipogenesis in the liver and the adipose tissue is more than doubled. Hyperglycemia and markedly elevated plasma insulin concentration are associated with an increase in the number and size of the beta cells of the islets of Langerhans [23]. Moreover, the secretion of glucagon is also enhanced. Another feature is the impaired immune response with defective cell-mediated immunity and lymphoid atrophy [24].

Diabetic mice db/db have a mutation that inactivates leptin receptor. The phenotype of the homozygous mice includes obesity, insulin resistance, and diabetes. These mice are polyphagic, polydipsic, and polyuric [23]. An impaired cell-related immune response is also seen [24].

Also, Zucker Diabetic fatty rats (fa/fa rats) serve as a model of early-onset diabetes. Analogously to the db/db mouse, fa/fa rats have a mutation in the leptin receptor. They are hyperglycemic, hyperinsulinemic, and hypertriglyceridemic and they present with not only moderately increased blood pressure but also increased serum markers of inflammation [23].

## The impact of leptin on skin and hair

Although adipocytes of white tissue are the prevalent site of leptin synthesis, it has been reported that fibroblasts and keratinocytes also possess the ability to synthesize leptin and express its receptors [4, 25–27]. The expression of leptin receptors has been detected in the epidermis, predominantly in the basal layer, and in the hair follicle papilla cells [4, 25, 26]. Taking together, these findings suggest not only a central action but also an autocrine and paracrine action of leptin.

Interestingly, leptin possesses the ability to stimulate the proliferation of keratinocytes and fibroblasts, epithelialization, and collagen synthesis. These mechanisms lead to an improvement in skin regeneration [25–28]. Moreover, local synthesis and secretion of leptin increase after skin injury and result in shortening the period of wound healing [29]. Leptin also supports skin microorganism defense by activation of the expression of human defensine 2 [30]. However, by activation of the STAT3 signaling pathway, leptin can trigger proliferation, differentiation, migration, and stabilization of cells in the skin as well as it may modulate angiogenesis [28, 29]. Lee et al. investigated the molecular mechanism of leptin impact on keratinocytes by observation genome-wide transcriptional responses of normal human keratinocytes (NHKs) [25] Leptin enhanced intracellular signaling and induced pro-inflammatory reaction in keratinocytes, by increasing the production of interleukins in a similar mechanism as it is observed in immune cells. Above mentioned leptin action might have a significant impact on the pathomechanism of skin diseases connected with obesity [25–31]. With a high probability, leptin derived centrally as well as in paracrine or even autocrine way can affect the skin under both physiological and pathological conditions.

Immunohistochemical analyses revealed the presence of leptin protein and leptin mRNA in the hair structures including the matrix, inner root sheath, and the follicular dermal papilla [32–34]. Noticeably, in transgenic mice with leptin receptor deficiency (db/db), anagen was found to be delayed [32]. The group of Sumikawa suggested that leptin could be an essential anagen activator in the second hair cycle and might prompt hair growth [32]. Besides, the injection of exogenous leptin stimulated anagen conversion in resting hair follicles [34]. Nevertheless, the exact mechanism of leptin’s influence on the hair cycle and hair growth is still not fully elucidated [32–34].

**Fig. 1:**
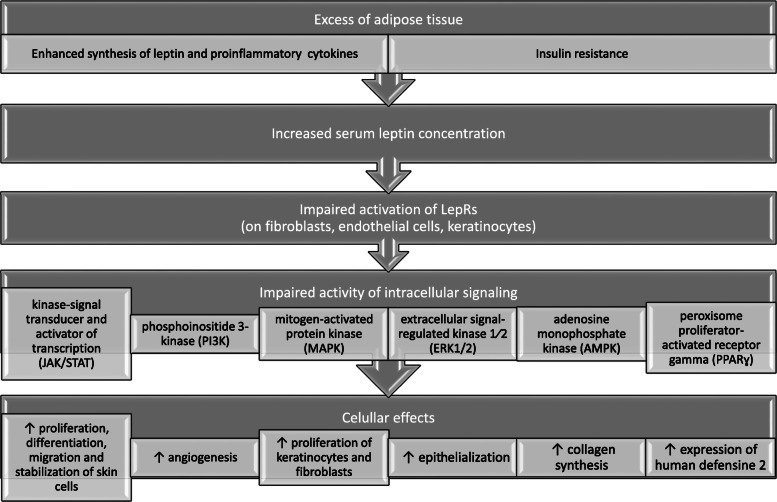
The suggested mechanisms of leptin activity in the skin

The suggested mechanisms of leptin activity in the skin are presented in Fig. 1.

## Leptin and psoriasis

Psoriasis is one of the most common skin diseases with a multifactorial pathogenesis. The prevalence is various in different regions, but overall it reaches 2% of the world population [35]. The difference in incidence rate is strongly linked to the development status with a higher number observed in high-developed countries.

The skin inflammation process is underlying skin changes and usually has three features: erythema, thickening, and scale. Some typical features are observed in the histological examination of psoriatic skin:

1. epidermal acanthosis, hyperkeratosis, and parakeratosis
2. dilated capillaries in the papillary dermis
3. mixed inflammatory infiltration [35].

Psoriasis is considered to be a kind of genetic-, autoimmune-, and metabolic-derived disease. It has been speculated that when the genetically predisposed individual is exposed to specific environmental factors (EFs) that act along with epigenetic alternations, he may develop psoriatic skin changes [35–41]. The well-known EFs that are strongly linked to psoriasis include dietary habits along with obesity, microbiota, infections, alcohol intake, tobacco smoking, and psychological factors [42–44].

There are numerous studies concerning adipokine levels in a course of psoriasis, and these observations comprised not only leptin but also resistin, adiponectin, and others [45–52]. Higher levels of leptin in sera of psoriatic patients in comparison with the controls were observed [53–56]. Besides, a marked increase of leptin was found in obese individuals, particularly in those with the coexistence of psoriasis and obesity [48, 49]. Moreover, Mitsuyama et al. indicated that leptin mRNA expression was significantly enhanced in subcutaneous adipose tissue (SAT) of obese psoriatic individuals as compared to the results of non-obese counterparts. Furthermore, both leptin levels and leptin mRNA expression in the SAT of non-obese psoriatic patients did not differ significantly from non-obese non-psoriatic controls [48]. Of note, some studies indicated a decrease in peripheral levels of leptin after systemic therapy of psoriasis [50–52]. A positive correlation between serum leptin concentration and the severity of psoriasis lesions, evaluated with the Psoriasis Area and Severity Index (PASI), was also found [53]. Moreover, immunological changes were seen in psoriasis including that levels of pro-inflammatory cytokines produced by the Th1 lymphocyte subtype were greater than the concentration of compounds secreted by the Th2 subtype [57]. Due to its pro-inflammatory activity, leptin promoted IL-1, IL-6, chemokine 8 (CXCL8), and TNF-α production, which also impacts psoriasis [45–47, 58]. All the above-mentioned processes stimulate the Th1/Th17 axis resulting in a higher concentration of IL-17/IL-23 [45–47, 58–60]. Besides, activation of this axis being previously enhanced by the JAK/STAT3 pathway, in which leptin is involved, can also exaggerate angiogenesis seen in psoriatic lesions [61].

It is of high importance that the analysis of psoriatic patient’s skin showed a high expression of leptin in cell plasma within all layers of the epidermis, and the presence of many inflammatory cells in contrast to the healthy skin, where leptin is present strictly in the basal layer cells of the epidermis. Greater expression of leptin and leptin receptor was observed in the epidermis of individuals with a severe form of psoriasis in comparison to the patients with mild to moderate form of the disease [53]. It has been suggested that enhanced levels of leptin in the patient’s skin may induce the production of amphiregulin, which is the epidermal growth factor protein with a probable role in keratinocyte proliferation [58]. Furthermore, the concentration of chemokine inducing the keratinocyte proliferation, C-X-C motif ligand 8 (CXCL8), was also higher in the skin of psoriatic patients [58]. It is also worth noting that leptin may stimulate CXCL8 production by monocytes [58].

Studies on animal models broaden our knowledge of the potential role of leptin in psoriasis. Stjerholm T. et al. revealed that ob/ob mice responded to imiquimod, psoriasis inducing factor, differently to the controls. In ob/ob mice the imiquimod administration to the skin resulted in attenuation of clinical signs as well as decreased mRNA expression of IL-17 and IL-22 in dorsal skin [24]. The group of Kanemaru demonstrated exacerbation of several psoriatic features including ear swelling and epidermal thickness, and increased expression of IL- 17A and IL- 22 mRNA in imiquimod-treated skin of db/db mice as compared to control. Interestingly, in the db/db model other psoriatic parameters were not aggravated. In detail, angiogenesis was inhibited, and the number of dendritic epidermal T cells was decreased [62]. Noticeably, the results reported by Nakamizo and co-workers indicated that the IL-17a expression and number of γδ TCRmid γδ T cells in the skin were comparable between those of ob/ob mice and control lean mice, whereas obese mice fed with a high-fat diet exhibited increased accumulation of IL-17A-producing Vγ4+ γδ T cells in the skin and aggravated psoriatic dermatitis [63]. Therefore, it could be speculated that the severity of psoriatic lesions is mostly associated with increased inflammation in the course of obesity that could partially be related to leptin activity rather than be a direct effect of enhanced leptin levels.

Moreover, there are in vitro studies evaluating the influence of leptin stimulation or leptin administration on normal human epidermal keratinocytes. An increase in proliferation and production of pro-inflammatory proteins was observed [24, 28, 64].

Finally, there is still little attention paid to the leptin gene polymorphism in a course of psoriasis. The group of Torres did not reveal any significant differences in leptin gene polymorphism of rs2167270(19 G/A), rs1137100(326 A/G), and adiponectin gene polymorphism rs1501299(276 G/T), as well as any differences in leptin levels, development of arteriosclerosis and adiposity in their cohort of psoriatic patients [65]. Karpouzis et al. who examined leptin gene polymorphism rs2060713 did not establish any link with psoriasis [66]. In contrast, Abdel Hay et al. suggested that leptin gene G2548A polymorphism could be a predictor of higher plasma leptin and increased risk of psoriasis [67].

## Leptin in other skin diseases

Apart from the disfunction of anagen activation, the lack of activation of the STAT3 pathway leads to fibrosis of the dermis, atrophy of the subcutaneous tissue, and the higher concentration of inflammatory cells in the subcutaneous tissue [16, 33]. Therefore, the question could be raised whether there is a link between insufficient leptin signaling pathways and skin fibrosis diseases. Several meta-analyses showed that in patients suffering from systemic sclerosis (SSc) leptin levels were comparable to those of the healthy population. However, decreased leptin concentration in serum was observed in active SSc patients when compared to the results of inactive SSc individuals. Consequently, these results suggest that leptin might be a potential activity marker of disease severity [22, 68–70]. Nevertheless, the role of adipokines in SSc requires further intensive research.

Recently, a possible role of leptin in systemic lupus erythematosus (SLE) pathogenesis has been emphasized. In mice models, it was shown that leptin via the RAR-related orphan receptor gamma (RORγ) promoted differentiation of lymphocyte Th17 subtype and enhanced synthesis of IL-17 [71]. Leptin, together with another pro-inflammatory factor, neutrophil-activating protein (NAP-2), activated the PI3k/Akt pathway in SLE patients, causing aging of the mesenchymal stem cells [72]. Moreover, leptin promoted survival and proliferation of auto-reactive T lymphocytes in mice with SLE-like mutation (NZBxNZW)F1 [73]. The inhibiting effect of leptin on the T regulatory subtype (Treg) was also suggested [22, 74]. Leptin may influence the population of immunocyte subsets as well as may modulate cytokine secretion, promote anti-apoptotic protein expression and prolong exposure to self-antigens leading to an enhancement of the autoimmune response but the interaction between leptin and SLE is currently not fully understood [75]. Besides, the results of studies regarding serum levels of leptin in patients with SLE in comparison with the controls are inconsistent. Additionally, no significant differences were observed between patients with the active and inactive phase of the disease [75–82]. Nevertheless, a lower leptin concentration was found in the concomitance of joint inflammation and neurological symptoms in a course of SLE [79].

Importantly, obesity and excess of adipose tissue result in higher leptin levels and increase the risk of many tumors including skin tumors, melanoma, and non-pigment tumors [83–87]. Melanoma is considered to be an obesity-related malignancy [88]. According to the literature, increased leptin concentration may accelerate the growth of melanoma [85–87]. Noticeably, the in vitro and in vivo study on mice model conducted by Malvi et al. revealed that controlling body weight either by pharmacological intervention or by dietary restriction may diminish the rapid progression of melanoma. There is a possibility that weight reduction might slow down obesity-promoted tumor progression by normalizing serum levels of adipokines, which affect tumor-promoting molecules and signaling pathways [88].

Furthermore, obesity has also been reported to influence therapeutic responses in some types of cancers. It has been speculated that the leptin pathways are supposed to mediate the obesity-associated impairment of chemotherapeutic responses. The group of Malvi showed that leptin administration may impair the efficacy of dacarbazine therapy of melanoma in obese ob/ob and db/db mice models [89]. Additionally, Chi et al. suggested that adipocyte-derived molecules may be responsible for the resistance of melanoma cells to chemotherapeutic drugs and agents targeting the PI3K/Akt and MEK/ERK pathways. The authors assumed that inhibition of the leptin/ LepR system could enhance the efficacy of multiple therapeutic approaches in the therapy of melanoma [90]. The researchers also reported that increased serum leptin levels may have an impact on melanoma progression and predict sentinel node (SN) metastasis. The serum level of leptin was significantly higher in the SN-positive group compared to the SN-negative group of melanoma subjects [91]. Therefore, taking into account the results of the above-mentioned studies, it could be indicated that controlling obesity and reducing leptin levels may be advantageous in patients with melanoma. Data considering the association between leptin and non-melanoma skin cancers are scanty. The immunohistochemical study revealed that the expression of leptin by tumor and stromal cells of squamous cell carcinoma may contribute to its progression by promoting angiogenesis with subsequently acquiring large tumor size and then advanced stage. Conversely, the expression of leptin in basal cell carcinoma was very limited and reflected the irrelevant role of obesity in the induction of this kind of skin cancer [92].

Of note, recent research has shown a positive correlation between serum leptin levels and the number of skin tags. Furthermore, a high level of leptin and impaired leptin receptors on keratinocytes and fibroblasts can trigger cell proliferation and differentiation into skin tag lesions [93–96].

Moreover, it has been found that obesity is a risk factor of hidradenitis suppurativa (HS) development, and patients with HS suffer from obesity and metabolic syndrome more often than the controls. Malara and colleagues observed that leptin was significantly increased in patients with HS. It should be emphasized that enhanced local concentration of leptin in subcutaneous adipose tissue caused an intensification of inflammatory processes in the skin of patients with HS and, additionally, resulted in an increase of systemic inflammation. Both phenomena might exacerbate the symptoms of HS [97, 98].

The potential role of leptin in acne vulgaris is still under discussion. There is a limited number of studies evaluating leptin in acne vulgaris. It is accepted that acne is a chronic inflammatory disease of the pilosebaceous with multifactorial pathogenesis including hyperseborrhoea, abnormal follicular keratinization, and *Propionibacterium acnes* colonization [99, 100]. The production of sebum is activated by various receptors, including leptin receptors, expressed in the sebaceous gland. The function of leptin in the sebocyte is to form lipid droplets within the cell. Moreover, leptin can activate the STAT-3 and NF-κB pathways and induce pro-inflammatory enzyme and cytokine (IL-6 and IL-8) secretion in human sebocytes, which suggests that the leptin signaling may be involved in the pro-inflammatory regulation of sebaceous lipid metabolism. Given the fact that secretion of leptin is a response to increased lipid uptake, leptin might be regarded as a link between improper diet and the development of inflammatory acne [100, 101]. However, investigations of the association between serum level of leptin and acne vulgaris revealed no statistically significant differences between serum leptin levels in patients with acne vulgaris and healthy controls [102–104].

Regardless of the possible impact of leptin on the hair follicle, the role of this adipokine in hair loss remains unknown. The association between obesity, leptin, and androgenetic alopecia (AGA) in males has been investigated by the group of Yang [105, 106]. Higher BMI was significantly correlated with increased severity of hair loss in men with male-pattern AGA, particularly in those with early-onset of AGA. Moreover, the serum level of leptin was higher in AGA subjects compared to non-AGA subjects, but the leptin level was no associated with the severity of the disease. These findings might suggest that enhanced plasma leptin levels could be related to a higher risk of developing AGA in men and leptin could play a role in the pathogenesis of AGA. However, the relationship between leptin and AGA requires further investigation.

The epidemiologic studies indicated an association between obesity and the prevalence and severity of atopic dermatitis (AD) [107–109]. Besides, prolonged obesity in early childhood is considered to be a risk factor for developing AD. As the pro-inflammatory effect of leptin is widely known, the researchers aimed to investigate serum leptin levels in AD. Interestingly, studies showed the differences in leptin levels according to types of dermatitis, IgE-mediated and non-IgE mediated atopic dermatitis. The group of Han revealed no significant differences in the analysis of leptin levels according to the SCORAD (scoring atopic dermatitis) index, which had been used to evaluate the severity of disease [110]. Also, the study of Seo et al. that comprised Korean’s children population, showed that leptin levels were elevated in children with non-atopic AD compared to those subjects with atopic AD, and leptin levels were inversely correlated with the severity of AD. The fact that leptin enhanced non-atopic Th1 immune response and there was no association between serum leptin and atopic sensitization suggest that the role of the leptin pathway in developing AD may involve non-IgE mediated mechanisms [111].

Other authors did not observe significant differences between leptin levels in subjects with and without AD [112, 113].

The potential role of leptin in selected skin diseases is presented in Table 1.

**Table 1:** The potential role of leptin in selected skin diseases

Skin disease	Serum leptin level	Potential effect	References
Psoriasis	↑	- proinflammatory: promotion of Th1/Th17 axis (↑IL-1, IL-6, IL-17/23, ↑CXCL8, ↑TNF-α) - angiogenesis: ↑JAK/STAT3 pathway - keratinocytegenesis: ↑amphiregulin, ↑CXCL8	[45–48, 52–56, 58]
Systemic sclerosis	no change or ↓	- antifibrotic: ↓JAK/STAT3 pathway	[22, 68–70]
Systemic lupus erythematosus	↑	- proinflammatory: promotion of the Th1/Th17 axis, inhibition of Treg - aging of mesenchymal stem cells (via the NAP-2 and PI3K-Akt pathway)	[22, 71–82]
Melanoma and non-pigment tumors	↑	- angiogenesis: ↑JAK/STAT3 pathway, ↑VEGF - mitogenic	[83–87]
Skin tags	↑	- ↑keratinocyte and fibroblast growth	[93–96]
Hidradenitis suppurativa	↑	- proinflammatory: promotion of Th1	[97, 98]
Acne vulgaris	no change	- pro-inflammatory regulation of sebaceous lipid metabolism	[102–104]
Male androgenetic alopecia	↑	- unknown role	[105, 106]
Atopic dermatitis	no change	- ↑non-atopic th1 immune response	[112, 113]

## Conclusions and perspectives

Leptin is a pluripotent adipokine and its activity has been associated with the development and maintenance of pro-inflammatory immune responses. The effects of leptin on the processes taking place within the skin and hair, as well as its role in the pathogenesis of different skin diseases, have been confirmed. As a consequence of adiposity and enhanced leptin levels, especially in conjunction with a reduced response of leptin receptors along with dysregulation of signaling pathways, several pathological processes occur in the skin and skin appendages. Although mechanisms of leptin activity have been thoroughly studied, the exact role of leptin in skin disorders still needs further investigations. However, when considering the relationship between leptin and skin, it should be taken into account that not only increased leptin levels have a negative impact but also probably a whole spectrum of adipokines and cytokines related to low-grade inflammation might be responsible for the malfunction of the skin. Then, it is a challenge to find all of the potential confounding factors influencing the skin pathology. Moreover, it is also important to find the complex interrelation between those compounds as well as to find the exact mechanism of their action within the skin and skin appendages. It needs to be highlighted that no all activities of leptin in the skin nor the detailed role of leptin in skin disease are fully understood. Another unsolved problem is that there is no clear explanation of why some obese individuals present a worse course of skin diseases. Besides, it is still an open question of whether pharmacological intervention resulting in decreasing leptin secretion or breaking the leptin receptor resistance could be a target in different skin disease treatment. However, it seems that the restoration of leptin receptor sensitivity is the most important strategy in treating obese-related diseases. Firstly, reducing central, hypothalamic, endoplasmic reticulum stress could be an approach to improve leptin resistance [114]. Furthermore, several emerging leptin sensitizers acting on the different points of leptin signaling are under investigation. Amongst these compounds, they are plant-derived substances (e.g. betulinic acid, Withaferin-A, celastrol, or KBH-1), chitosan oligosaccharide, neuropeptide oxytocin, glucagon-like peptide-1 (GLP1) analog (liraglutide) and others [114, 115].

Finally, taking into account that there is no accessible leptin-oriented medication, the proper treatment of obesity could result in better skin condition. Up to date, the most important recommendation is to prevent and treat obesity.

## Data Availability

not applicable.

## Abbreviations

AD: Atopic dermatitis
AGA: Androgenetic alopecia
AMPK: Adenosine monophosphate kinase
BMI: Body mass index
CXCL8: C-X-C motif ligand 8
EFs: Environmental factors
ERK1/2: Extracellular signal-regulated kinase 1/2
HS: Hidradenitis suppurativa
IL: Interleukin
JAKs: Janus kinases
LepRs: Leptin receptors
MAPK: Mitogen-activated protein kinase
mRNA: Messenger ribonucleic acid
NAP-2: Neutrophil-activating protein 2
NHKs: Normal human keratinocytes
NK: Natural killer
NO: Nitric oxide
PASI: Psoriasis Area and Severity Index
PGC /PPAR: PPAR gamma coactivator/peroxisome proliferator-activated receptor
PI3K: Phosphoinositide 3-kinase
RORγ: RAR-related orphan receptor gamma
ROS: Reactive oxygen species
SAT: Subcutaneous adipose tissue
SLE: Systemic lupus erythematosus
SN: Sentinel node
sOBR: Soluble receptor
SOCS3: Suppressor of cytokine signaling 3
SSc: Systemic sclerosis
STATs: Signal transducers and activators of transcription
TNF-α: Tumor necrosis factor α
Treg: T regulatory subtype

## References

[1] González-Muniesa P, Mártinez-González MA, Hu FB, Després JP, Matsuzawa Y, Loos RJF, Moreno LA, Bray GA, Martinez JA (2017). Obesity. Nat Rev Dis Primers.

[2] Münzberg H, Morrison CD (2015). Structure, production and signaling of leptin. Metabolism.

[3] Denver RJ, Bonett RM, Boorse GC (2011). Evolution of leptin structure and function. Neuroendocrinology.

[4] Poeggeler B, Schulz C, Pappolla MA, Bodó E, Tiede S, Lehnert H, Paus R (2010). Leptin and the skin: a new frontier. Exp Dermatol.

[5] Kelesidis T, Kelesidis I, Chou S, Mantzoros CS (2010). Narrative review: the role of leptin in human physiology: emerging clinical applications. Ann Intern Med.

[6] Ünlü B, Türsen Ü (2018). Autoimmune skin diseases and the metabolic syndrome. Clin Dermatol.

[7] Saltiel AR, Olefsky JM (2017). Inflammatory mechanisms linking obesity and metabolic disease. J Clin Invest.

[8] Makki K, Froguel P, Wolowczuk I (2013). Adipose tissue in obesity-related inflammation and insulin resistance: cells, cytokines, and chemokines. ISRN Inflamm.

[9] Deng T, Lyon CJ, Bergin S, Caligiuri MA, Hsueh WA (2016). Obesity, inflammation, and Cancer. Annu Rev Pathol.

[10] Kuroda M, Sakaue H (2017). Adipocyte death and chronic inflammation in obesity. J Med Investig.

[11] Tobin AM, Ahern T, Rogers S, Collins P, O'Shea D, Kirby B (2013). The dermatological consequences of obesity. Int J Dermatol.

[12] Hirt PA, Castillo DE, Yosipovitch G, Keri JE (2019). Skin changes in the obese patient. J Am Acad Dermatol.

[13] Crujeiras AB, Carreira MC, Cabia B, Andrade S, Amil M, Casanueva FF (2015). Leptin resistance in obesity: an epigenetic landscape. Life Sci.

[14] Liu J, Yang X, Yu S, Zheng R (2018). The Leptin signaling. Adv Exp Med Biol.

[15] Wauman J, Zabeau L, Tavernier J (2017). The Leptin receptor complex: heavier than expected?. Front Endocrinol (Lausanne).

[16] Bates SH, Stearns WH, Dundon TA, Schubert M, Tso AW, Wang Y, Banks AS, Lavery HJ, Haq AK, Maratos-Flier E (2003). STAT3 signalling is required for leptin regulation of energy balance but not reproduction. Nature.

[17] Schaab M, Kratzsch J (2015). The soluble leptin receptor. Best Pract Res Clin Endocrinol Metab.

[18] Kwon O, Kim KW, Kim MS (2016). Leptin signalling pathways in hypothalamic neurons. Cell Mol Life Sci.

[19] Luo GF, Yu TY, Wen XH, Li Y, Yang GS (2008). Alteration of mitochondrial oxidative capacity during porcine preadipocyte differentiation and in response to leptin. Mol Cell Biochem.

[20] La Cava A (2017). Leptin in inflammation and autoimmunity. Cytokine.

[21] Pérez-Pérez A, Vilariño-García T, Fernández-Riejos P, Martín-González J, Segura-Egea JJ, Sánchez-Margalet V (2017). Role of leptin as a link between metabolism and the immune system. Cytokine Growth Factor Rev.

[22] Navarini L, Margiotta DPE, Vadacca M, Afeltra A (2018). Leptin in autoimmune mechanisms of systemic rheumatic diseases. Cancer Lett.

[23] Ritskes-Hoitinga M, Tobin G, Jensen TL, Mikkelsen LF, Hedrich HJ (2012). Chapter 4.3 - Nutrition of the Laboratory Mouse. The Laboratory Mouse (Second Edition).

[24] Stjernholm T, Ommen P, Langkilde A, Johansen C, Iversen L, Rosada C, Stenderup K (2017). Leptin deficiency in mice counteracts imiquimod (IMQ)-induced psoriasis-like skin inflammation while leptin stimulation induces inflammation in human keratinocytes. Exp Dermatol.

[25] Lee M, Lee E, Jin SH, Ahn S, Kim SO, Kim J, Choi D, Lim KM, Lee ST, Noh M (2018). Leptin regulates the pro-inflammatory response in human epidermal keratinocytes. Arch Dermatol Res.

[26] Murad A, Nath AK, Cha ST, Demir E, Flores-Riveros J, Sierra-Honigmann MR (2003). Leptin is an autocrine/paracrine regulator of wound healing. FASEB J.

[27] Glasow A, Kiess W, Anderegg U, Berthold A, Bottner A, Kratzsch J (2001). Expression of leptin (Ob) and leptin receptor (Ob-R) in human fibroblasts: regulation of leptin secretion by insulin. J Clin Endocrinol Metab.

[28] Frank S, Stallmeyer B, Kämpfer H, Kolb N, Pfeilschifter J (2000). Leptin enhances wound re-epithelialization and constitutes a direct function of leptin in skin repair. J Clin Invest.

[29] Tadokoro S, Ide S, Tokuyama R, Umeki H, Tatehara S, Kataoka S, Satomura K (2015). Leptin promotes wound healing in the skin. PLoS One.

[30] Kanda N, Watanabe S (2008). Leptin enhances human beta-defensin-2 production in human keratinocytes. Endocrinology.

[31] Tong KM, Shieh DC, Chen CP, Tzeng CY, Wang SP, Huang KC, Chiu YC, Fong YC, Tang CH (2008). Leptin induces IL-8 expression via leptin receptor, IRS-1, PI3K, Akt cascade and promotion of NF-kappaB/p300 binding in human synovial fibroblasts. Cell Signal.

[32] Sumikawa Y, Inui S, Nakajima T, Itami S (2014). Hair cycle control by leptin as a new anagen inducer. Exp Dermatol.

[33] Watabe R, Yamaguchi T, Kabashima-Kubo R, Yoshioka M, Nishio D, Nakamura M (2014). Leptin controls hair follicle cycling. Exp Dermatol.

[34] Won CH, Yoo HG, Kwon OS, Sung MY, Kang YJ, Chung JH, Park BS, Sung JH, Kim WS, Kim KH (2010). Hair growth promoting effects of adipose tissue-derived stem cells. In J Dermatol Sci.

[35] Lebwohl MG, Bachelez H, Barker J, Girolomoni G, Kavanaugh A, Langley RG, Paul CF, Puig L, Reich K, van de Kerkhof PC (2014). Patient perspectives in the management of psoriasis: results from the population-based Multinational Assessment of Psoriasis and Psoriatic Arthritis Survey. J Am Acad Dermatol.

[36] Russell TJ, Schultes LM, Kuban DJ (1972). Histocompatibility (HL-A) antigens associated with psoriasis. N Engl J Med.

[37] Roszkiewicz M, Dopytalska K, Szymańska E, Jakimiuk A, Walecka I. Environmental risk factors and epigenetic alternations in psoriasis. Ann Agric Environ Med. 2020;27:335–42. 10.26444/aaem/112107 32955211

[38] Schmitt-Egenolf M, Eiermann TH, Boehncke WH, Ständer M, Sterry W (1996). Familial juvenile onset psoriasis is associated with the human leukocyte antigen (HLA) class I side of the extended haplotype Cw6-B57-DRB1*0701-DQA1*0201-DQB1*0303: a population- and family-based study. J Invest Dermatol.

[39] Barrea L, Nappi F, Di Somma C, Savanelli M, Falco A, Balato A, Balato N, Savastano S (2016). Environmental risk factors in psoriasis: the point of view of the nutritionist. Int J Environ Res Public Health.

[40] Gervin K, Vigeland MD, Mattingsdal M, Hammerø M, Nygård H, Olsen AO, Brandt I, Harris JR, Undlien DE, Lyle R (2012). DNA methylation and gene expression changes in monozygotic twins discordant for psoriasis: identification of epigenetically dysregulated genes. PLoS Genet.

[41] Nair RP, Duffin KC, Helms C, Ding J, Stuart PE, Goldgar D, Gudjonsson JE, Li Y, Tejasvi T, Feng BJ (2009). Genome-wide scan reveals association of psoriasis with IL-23 and NF-kappaB pathways. Nat Genet.

[42] Zeng J, Luo S, Huang Y, Lu Q (2017). Critical role of environmental factors in the pathogenesis of psoriasis. J Dermatol.

[43] Naldi L, Chatenoud L, Linder D, Belloni Fortina A, Peserico A, Virgili AR, Bruni PL, Ingordo V, Lo Scocco G, Solaroli C (2005). Cigarette smoking, body mass index, and stressful life events as risk factors for psoriasis: results from an Italian case-control study. J Invest Dermatol.

[44] Rachakonda TD, Dhillon JS, Florek AG, Armstrong AW (2015). Effect of tonsillectomy on psoriasis: a systematic review. J Am Acad Dermatol.

[45] Nakajima H, Nakajima K, Tarutani M, Sano S (2013). Clear association between serum levels of adipokines and T-helper 17-related cytokines in patients with psoriasis. Clin Exp Dermatol.

[46] Xue K, Liu H, Jian Q, Liu B, Zhu D, Zhang M, Gao L, Li C (2013). Leptin induces secretion of pro-inflammatory cytokines by human keratinocytes in vitro--a possible reason for increased severity of psoriasis in patients with a high body mass index. Exp Dermatol.

[47] Zhu KJ, Zhang C, Li M, Zhu CY, Shi G, Fan YM (2013). Leptin levels in patients with psoriasis: a meta-analysis. Clin Exp Dermatol.

[48] Mitsuyama S, Abe F, Kimura M, Yoshida M, Higuchi T (2015). Association between leptin gene expression in subcutaneous adipose tissue and circulating leptin levels in obese patients with psoriasis. Arch Dermatol Res.

[49] El-Boghdady NA, Ismail MF, Abd-Alhameed MF, Ahmed AS, Ahmed HH (2018). Bidirectional association between psoriasis and obesity: benefits and risks. J Interf Cytokine Res.

[50] Campanati A, Ganzetti G, Giuliodori K, Marra M, Bonfigli A, Testa R, Offidani A (2015). Serum levels of adipocytokines in psoriasis patients receiving tumor necrosis factor-α inhibitors: results of a retrospective analysis. Int J Dermatol.

[51] Takahashi H, Tsuji H, Ishida-Yamamoto A, Iizuka H (2013). Serum level of adiponectin increases and those of leptin and resistin decrease following the treatment of psoriasis. J Dermatol.

[52] Ozdemir M, Yüksel M, Gökbel H, Okudan N, Mevlitoğlu I (2012). Serum leptin, adiponectin, resistin and ghrelin levels in psoriatic patients treated with cyclosporin. J Dermatol.

[53] Cerman AA, Bozkurt S, Sav A, Tulunay A, Elbaşi MO, Ergun T (2008). Serum leptin levels, skin leptin and leptin receptor expression in psoriasis. Br J Dermatol.

[54] Wang Y, Chen J, Zhao Y, Geng L, Song F, Chen HD (2008). Psoriasis is associated with increased levels of serum leptin. In Br J Dermatol.

[55] Coimbra S, Oliveira H, Reis F, Belo L, Rocha S, Quintanilha A, Figueiredo A, Teixeira F, Castro E, Rocha-Pereira P, Santos-Silva A (2010). Circulating adipokine levels in Portuguese patients with psoriasis vulgaris according to body mass index, severity and therapy. J Eur Acad Dermatol Venereol.

[56] Kyriakou A, Patsatsi A, Sotiriadis D, Goulis DG (2017). Serum Leptin, Resistin, and Adiponectin concentrations in psoriasis: a meta-analysis of observational studies. Dermatology.

[57] Eberle F, Brück J, Holstein J, Hirahara K, Ghoreschi K (2016). Recent advances in understanding psoriasis. F1000Research.

[58] Johnston A, Arnadottir S, Gudjonsson JE, Aphale A, Sigmarsdottir AA, Gunnarsson SI, Steinsson JT, Elder JT, Valdimarsson H (2008). Obesity in psoriasis: leptin and resistin as mediators of cutaneous inflammation. Br J Dermatol.

[59] Fritz Y, Klenotic PA, Swindell WR, Yin ZQ, Groft SG, Zhang L, Baliwag J, Camhi MI, Diaconu D, Young AB (2017). Induction of alternative Proinflammatory cytokines accounts for sustained Psoriasiform skin inflammation in IL-17C+IL-6KO mice. J Invest Dermatol.

[60] Grossman RM, Krueger J, Yourish D, Granelli-Piperno A, Murphy DP, May LT, Kupper TS, Sehgal PB, Gottlieb AB (1989). Interleukin 6 is expressed in high levels in psoriatic skin and stimulates proliferation of cultured human keratinocytes. Proc Natl Acad Sci U S A.

[61] Calautti E, Avalle L, Poli V (2018). Psoriasis: a STAT3-centric view. Int J Mol Sci.

[62] Kanemaru K, Matsuyuki A, Nakamura Y, Fukami K (2015). Obesity exacerbates imiquimod-induced psoriasis-like epidermal hyperplasia and interleukin-17 and interleukin-22 production in mice. Exp Dermatol.

[63] Nakamizo S, Honda T, Adachi A, Nagatake T, Kunisawa J, Kitoh A, Otsuka A, Dainichi T, Nomura T, Ginhoux F (2017). High fat diet exacerbates murine psoriatic dermatitis by increasing the number of IL-17-producing γδ T cells. Sci Rep.

[64] Takahashi H, Honma M, Ishida-Yamamoto A, Iizuka H (2010). Adiponectin and leptin modulate cell proliferation and cytokine secretion of normal human keratinocytes and T lymphocytes. In J Dermatol Sci.

[65] Torres T, Bettencourt N, Ferreira J, Carvalho C, Mendonça D, Vasconcelos C, Selores M, Silva B (2015). Lack of association between leptin, leptin receptor, adiponectin gene polymorphisms and epicardial adipose tissue, abdominal visceral fat volume and atherosclerotic burden in psoriasis patients. Arch Physiol Biochem.

[66] Karpouzis A, Tripsianis G, Gatzidou E, Veletza S (2014). Assessment of Leptin gene polymorphism rs2060713 in psoriasis vulgaris. ISRN Dermatol.

[67] Abdel Hay RM, Rashed LA (2011). Association between the leptin gene 2548G/a polymorphism, the plasma leptin and the metabolic syndrome with psoriasis. Exp Dermatol.

[68] Zhao JH, Huang XL, Duan Y, Wang YJ, Chen SY, Wang J (2017). Serum adipokines levels in patients with systemic sclerosis: a meta-analysis. Mod Rheumatol.

[69] Lee YH, Song GG (2017). Meta-analysis of circulating adiponectin, leptin, and resistin levels in systemic sclerosis. Z Rheumatol.

[70] Budulgan M, Dilek B, Dağ ŞB, Batmaz I, Yıldız İ, Sarıyıldız MA, Çevik R, Nas K (2014). Relationship between serum leptin level and disease activity in patients with systemic sclerosis. Clin Rheumatol.

[71] Yu Y, Liu Y, Shi FD, Zou H, Matarese G, La Cava A (2013). Cutting edge: Leptin-induced RORγt expression in CD4+ T cells promotes Th17 responses in systemic lupus erythematosus. J Immunol.

[72] Chen H, Shi B, Feng X, Kong W, Chen W, Geng L, Chen J, Liu R, Li X, Gao X, Sun L (2015). Leptin and neutrophil-activating peptide 2 promote Mesenchymal stem cell senescence through activation of the phosphatidylinositol 3-kinase/Akt pathway in patients with systemic lupus Erythematosus. Arthritis Rheum.

[73] Amarilyo G, Iikuni N, Shi FD, Liu A, Matarese G, La Cava A (2013). Leptin promotes lupus T-cell autoimmunity. Clin Immunol.

[74] De Rosa V, Procaccini C, Cali G, Pirozzi G, Fontana S, Zappacosta S, La Cava A, Matarese G (2007). A key role of leptin in the control of regulatory T cell proliferation. Immunity.

[75] Yuan Q, Chen H, Li X, Wei J (2020). Leptin: an unappreciated key player in SLE. Clin Rheumatol.

[76] Al M, Ng L, Tyrrell P, Bargman J, Bradley T, Silverman E (2009). Adipokines as novel biomarkers in paediatric systemic lupus erythematosus. Rheumatology (Oxford).

[77] Garcia-Gonzalez A, Gonzalez-Lopez L, Valera-Gonzalez IC, Cardona-Muñoz EG, Salazar-Paramo M, González-Ortiz M, Martínez-Abundis E, Gamez-Nava JI (2002). Serum leptin levels in women with systemic lupus erythematosus. Rheumatol Int.

[78] Barbosa Vde S, Francescantônio PL, Silva NA (2015). Leptin and adiponectin in patients with systemic lupus erythematosus: clinical and laboratory correlations. Rev Bras Reumatol.

[79] Wisłowska M, Rok M, Stepień K, Kuklo-Kowalska A (2008). Serum leptin in systemic lupus erythematosus. Rheumatol Int.

[80] Li HM, Zhang TP, Leng RX, Li XP, Li XM, Pan HF (2015). Plasma/serum Leptin levels in patients with systemic lupus Erythematosus: a meta-analysis. Arch Med Res.

[81] De Sanctis JB, Zabaleta M, Bianco NE, Garmendia JV, Rivas L (2009). Serum adipokine levels in patients with systemic lupus erythematosus. Autoimmunity.

[82] Mohammed S, Abdalla M, Ismaeil W, Sheta M. Serum leptin in systemic lupus erythematosus patients: its correlation with disease activity and some disease parameters. Egyp Rheumatol. 2018;40:23–7.

[83] Lee CH, Woo YC, Wang Y, Yeung CY, Xu A, Lam KS (2015). Obesity, adipokines and cancer: an update. Clin Endocrinol.

[84] Præstegaard C, Kjær SK, Christensen J, Tjønneland A, Halkjær J, Jensen A. Obesity and risks for malignant melanoma and non-melanoma skin cancer: results from a large Danish prospective cohort study. J Invest Dermatol. 2015;135:901–4. 10.1038/jid.2014.438 25290686

[85] Gogas H, Trakatelli M, Dessypris N, Terzidis A, Katsambas A, Chrousos GP, Petridou ET (2008). Melanoma risk in association with serum leptin levels and lifestyle parameters: a case-control study. Ann Oncol.

[86] Brandon EL, Gu JW, Cantwell L, He Z, Wallace G, Hall JE (2009). Obesity promotes melanoma tumor growth: role of leptin. Cancer Biol Ther.

[87] Amjadi F, Mehdipoor R, Zarkesh-Esfahani H, Javanmard SH (2016). Leptin serves as angiogenic/mitogenic factor in melanoma tumor growth. Adv Biomed Res.

[88] Malvi P, Chaube B, Pandey V, Vijayakumar MV, Boreddy PR, Mohammad N, Singh SV, Bhat MK (2015). Obesity induced rapid melanoma progression is reversed by orlistat treatment and dietary intervention: role of adipokines. Mol Oncol.

[89] Malvi P, Chaube B, Singh SV, Mohammad N, Vijayakumar MV, Singh S, Chouhan S, Bhat MK (2018). Elevated circulatory levels of leptin and resistin impair therapeutic efficacy of dacarbazine in melanoma under obese state. Cancer Metab.

[90] Chi M, Chen J, Ye Y, Tseng HY, Lai F, Tay KH, Jin L, Guo ST, Jiang CC, Zhang XD (2014). Adipocytes contribute to resistance of human melanoma cells to chemotherapy and targeted therapy. Curr Med Chem.

[91] Oba J, Wei W, Gershenwald JE, Johnson MM, Wyatt CM, Ellerhorst JA, Grimm EA (2016). Elevated serum Leptin levels are associated with an increased risk of sentinel lymph node metastasis in cutaneous melanoma. Medicine (Baltimore).

[92] Farag AG, Elnaidany NF, El-Dien MM (2016). Immunohistochemical Expression of Leptin in Non Melanoma Skin Cancer. J Clin Diagn Res.

[93] El Safoury OS, Abdel Hay RM, Fawzy MM, Kadry D, Amin IM, Abu Zeid OM, Rashed LA (2011). Skin tags, leptin, metabolic syndrome and change of the life style. Indian J Dermatol Venereol Leprol.

[94] Putra IB, Siregar R, Jusuf NK, Ginting O, Nurhayati R (2019). Correlation between serum Leptin level with type and number of lesion skin tag. Open Access Maced J Med Sci.

[95] Gorpelioglu C, Erdal E, Ardicoglu Y, Adam B, Sarifakioglu E (2009). Serum leptin, atherogenic lipids and glucose levels in patients with skin tags. Indian J Dermatol.

[96] Shaheen MA, Abdel Fattah NS, Sayed YA, Saad AA (2012). Assessment of serum leptin, insulin resistance and metabolic syndrome in patients with skin tags. J Eur Acad Dermatol Venereol.

[97] Shalom G, Freud T, Harman-Boehm I, Polishchuk I, Cohen AD (2015). Hidradenitis suppurativa and metabolic syndrome: a comparative cross-sectional study of 3207 patients. Br J Dermatol.

[98] Malara A, Hughes R, Jennings L, Sweeney CM, Lynch M, Awdeh F, Timoney I, Tobin AM, Lynam-Loane K, Tobin L (2018). Adipokines are dysregulated in patients with hidradenitis suppurativa. Br J Dermatol.

[99] Moradi Tuchayi S, Makrantonaki E, Ganceviciene R, Dessinioti C, Feldman SR, Zouboulis CC (2015). Acne vulgaris. Nat Rev Dis Primers.

[100] Dréno B (2017). What is new in the pathophysiology of acne, an overview. J Eur Acad Dermatol Venereol.

[101] Törőcsik D, Kovács D, Camera E, Lovászi M, Cseri K, Nagy GG, Molinaro R, Rühl R, Tax G, Szabó K (2014). Leptin promotes a proinflammatory lipid profile and induces inflammatory pathways in human SZ95 sebocytes. Br J Dermatol.

[102] Chang HC, Lin MH, Huang YC (2019). Association between circulating adipokines and acne vulgaris: a systematic review and meta-analysis. Australas J Dermatol.

[103] Kaymak Y, Adisen E, Ilter N, Bideci A, Gurler D, Celik B (2007). Dietary glycemic index and glucose, insulin, insulin-like growth factor-I, insulin-like growth factor binding protein 3, and leptin levels in patients with acne. J Am Acad Dermatol.

[104] Ozuguz P, Kacar SD, Asik G, Ozuguz U, Karatas S. Evaluation of leptin, adiponectin, and ghrelin levels in patients with acne vulgaris. Hum Exp Toxicol. 2017;36:3–7. 10.1177/0960327116630355 26860691

[105] Yang CC, Hsieh FN, Lin LY, Hsu CK, Sheu HM, Chen W. Higher body mass index is associated with greater severity of alopecia in men with male-pattern androgenetic alopecia in Taiwan: a cross-sectional study. J Am Acad Dermatol. 2014;70:297–302 e291. 10.1016/j.jaad.2013.09.036 24184140

[106] Yang CC, Chung PL, Lin LY, Hughes MW, Tsai YS (2017). Higher plasma leptin is associated with higher risk of androgenetic alopecia in men. Exp Dermatol.

[107] Zhang A, Silverberg JI (2015). Association of atopic dermatitis with being overweight and obese: a systematic review and metaanalysis. J Am Acad Dermatol.

[108] Koutroulis I, Magnelli L, Gaughan J, Weiner E, Kratimenos P (2015). Atopic dermatitis is more severe in children over the age of two who have an increased body mass index. Acta Paediatr.

[109] Silverberg JI, Kleiman E, Lev-Tov H, Silverberg NB, Durkin HG, Joks R, Smith-Norowitz TA (2011). Association between obesity and atopic dermatitis in childhood: a case-control study. J Allergy Clin Immunol.

[110] Han B, Wu WH, Bae JM, Son SJ, Lee JH, Han TY (2016). Serum leptin and adiponectin levels in atopic dermatitis (AD) and their relation to disease severity. J Am Acad Dermatol.

[111] Seo S, Yoon WS, Cho Y, Park SH, Choung JT, Yoo Y (2016). Leptin and atopic dermatitis in Korean elementary school children. Iran J Allergy Asthma Immunol.

[112] Bostanci I, Atli O, Celebi N, Taşar A, Alpkarakoç E, Dallar Y (2004). Serum leptin level in children with atopic dermatitis-treated topical steroids. Pediatr Allergy Immunol.

[113] Balato N, Nino M, Patruno C, Matarese G, Ayala F (2011). "eczemas" and leptin. Dermatitis.

[114] Martínez-Sánchez N. There and back again: leptin actions in white adipose tissue. Int J Mol Sci. 2020;21:6039. 10.3390/ijms21176039 PMC7503240 32839413

[115] Delibasi T (2017). Future of obesity treatment: leptin sensitizers. Arch Gen Intern Med.

